# The epidemiology of postpartum malaria: a systematic review

**DOI:** 10.1186/1475-2875-11-114

**Published:** 2012-04-13

**Authors:** Machteld E Boel, Marcus J Rijken, Bernard J Brabin, François Nosten, Rose McGready

**Affiliations:** 1 Shoklo Malaria Research Unit, PO Box 46, 63110, Mae Sot Tak, Thailand; 2 Academic Medical Center, Meibergdreef 9, Postbus 22660, 1100 DD Amsterdam, The Netherlands; 3 Liverpool School of Tropical Medicine, Pembroke Place, Liverpool, Merseyside L3 5QA, UK; 4 Faculty of Tropical Medicine, Mahidol University, 420/6 Ratchawithi Road, Bangkok 10400, Thailand; 5 Centre for Tropical Medicine, Nuffield Department of Clinical Medicine, University of Oxford, CCVTM, Oxford OX3 7LJ, UK

**Keywords:** Malaria, Postpartum, Pregnancy, Delivery

## Abstract

Pregnant women are more susceptible to malaria than their non-pregnant counterparts. Less is known about the risk of malaria in the postpartum period. The epidemiology of postpartum malaria was systematically reviewed. Eleven articles fitted the inclusion criteria. Of the 10 studies that compared malaria data from the postpartum period with pregnancy data, nine studies suggested that the risk for malaria infection decreased after delivery. All three studies that compared postpartum data with non-pregnant non-postpartum women concluded that the risk did not return to pre-pregnancy levels immediately after delivery. The results of this review have to be carefully interpreted, as the majority of studies were not designed to study postpartum malaria, and there was large variability in study designs and reported outcomes. Current evidence suggests an effort should be made to detect and radically cure malaria during pregnancy so that women do not enter the postpartum period with residual parasites.

## Background

Pregnant women are more susceptible to malaria than their non-pregnant counterparts. This was described nearly a century ago[1], and applies to all malaria regions in the world, regardless of transmission intensity[2,3]. Primigravida tend to be more susceptible than multigravida, as women gain immunity against malaria during successive pregnancies, particularly in areas of high transmission[4].

To control *Plasmodium falciparum *malaria during pregnancy, the WHO Roll Back Malaria recommends several strategies: personal protection with insecticide impregnated bed nets (ITNs), intermittent preventive treatment (IPTp) and case management of anaemia and malaria illness with effective anti-malarial drugs[5]. A much less emphasized part of this recommendation is that women in the postpartum period should be encouraged to use ITNs. There are no other recommended preventive strategies for postpartum women. However, malaria was one of the leading causes of hospital admission and maternal death among postpartum women in Zambia[6] and India[7]. Little is known about the epidemiology and pathophysiology of postpartum malaria. In theory, the increased susceptibility during pregnancy normalizes immediately after delivery when the placenta, where the parasites can adhere during pregnancy, is expelled. This is supported by the reports of spontaneous clearance of *P. falciparum *parasites within 24 hours after delivery in high transmission areas in Africa[8,9]. In contrast, in Malawi[10] among women of which some were screened at day 0, others at day 1, 2, until 1 week after delivery, the proportion with a positive malaria blood smear was constant each day over the first seven days (K. Msyamboza, personal communication). This suggests that other factors may play a role, such as behavioural and physiological changes that attract mosquitoes during pregnancy[11], suppression of cell-mediated immunity and changes in maternal hormonal levels. These factors take much longer to normalize[12,13], and might extend beyond the usual limit of the postpartum period which is defined by WHO as the period until six weeks after delivery[14]. The primary objective of this systematic review was to describe the epidemiology of malaria in the postpartum period. This was achieved by comparing postpartum data: 1) to longitudinally collected malaria data from the same women during pregnancy; 2) to malaria data from non-pregnant, non-postpartum control women matched for age, area of residence and time; or 3) to both 1 and 2.

## Methods

References for this review were identified through searches of MEDLINE and the Malaria in Pregnancy library[15] with the terms 'malaria' AND 'postpartum' OR 'puerperium' using a combination of MeSH headings and keywords. The search was limited to humans, clinical trials, in English and Spanish language until 28 February 2011. Two authors independently performed eligibility assessment and, if disagreements were not resolved by consensus, a decision was made by a third author. The reporting guidance as described in the PRISMA statement was closely followed [16].

Criteria for inclusion were: a) laboratory confirmation of malaria in the postpartum period; b) clarity about the timing of malaria sampling; and c) similarity in epidemiological indicators to describe malaria in pregnant, postpartum and control women. Exclusion criteria were: a) selection bias e.g. only women with fever, b) seasonal bias e.g. longitudinal cohort study with enrolment only during one season in an area with seasonal transmission, c) unavailability of locally comparable data (pregnant or general population during the same time period) and d) less than two weeks follow up after delivery. If crucial data were missing, the authors were approached to provide additional information. Information extracted from each study included: (1) characteristics of the study (study subject, time period, location, enrolment criteria, length and frequency of follow up during pregnancy and postpartum, passive or active case detection, malaria species, parasite densities, symptoms, methods of diagnosing malaria, malaria endemicity and treatment) (2) quality of measures that could affect postpartum susceptibility (type of study, use of IPTp or chemoprophylaxis, samples taken during pregnancy or delivery, drug efficacy, treatment criteria, self-treatment with anti-malarials, use of control patients and percentage of lost to follow up) and (3) outcome measures (incidence, prevalence or rate ratios of malaria infection during pregnancy and postpartum and in non-pregnant non postpartum controls if available). To allow comparisons between different studies the outcome measures of each study were recalculated into proportions of infected women at certain time points during pregnancy, postpartum or in control women with confidence limits and p-values. In studies where all women were given chemoprophylaxis or IPTp, only the malaria data of the first antenatal visit were used for comparison with postpartum data. When IPTp or chemoprophylaxis was used in a randomized controlled trial, data of the placebo arm and the study drug arm were analysed separately.

## Results

**Figure 1:**
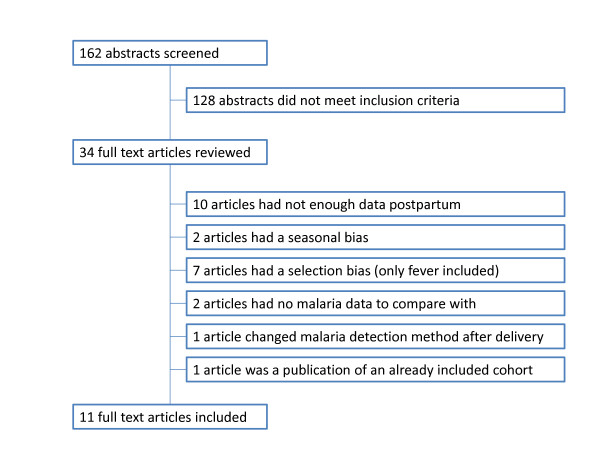
**Schematic draft of inclusion and exclusion process**.

The literature search provided a total of 162 abstracts of which 128 did not meet the inclusion criteria (Figure 1). The full text of the remaining 34 citations was examined in detail and of these, eleven studies met the inclusion criteria[17-27] (Figure 1). Two of these articles described the same population but used different methodology to detect malaria[23,25].

### Characteristics of the included studies

The eleven included studies were conducted between 1968 and 2005. Only three studies were designed to measure postpartum malaria[19,24,25]. The central research question of the eight remaining studies pertained to pregnancy related subjects (Additional file 1: Table S1), but the study follow-up was extended into the postpartum period for at least two months. All studies were conducted in highly endemic malarious areas: 10 studies were from Africa, where *P. falciparum *is the main specie of malaria and one in Papua New Guinea (PNG) where *Plasmodium vivax *is co-endemic with *P. falciparum *and *Plasmodium malariae*. These species were included in the analysis of the paper from PNG[17], but the analysis in the papers from Africa was restricted to *P.falciparum*, although three African studies mentioned *P. malariae *and/or *Plasmodium ovale *to occur occasionally[22,24,26].

### Quality measures of the included studies

**Figure 2:**
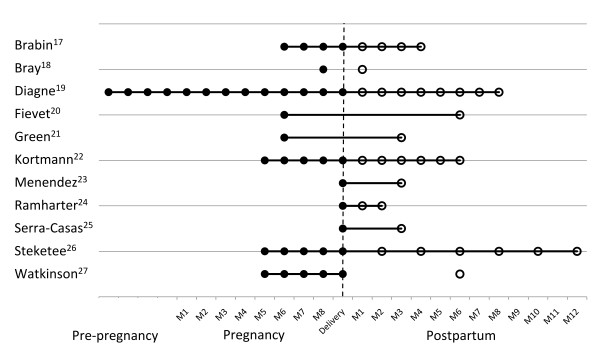
**Time scheme of sampling during pre-pregnancy, pregnancy or postpartum period**. M = month, D = delivery. Open circles (○) are used for the postpartum period, filled circles (●) for the period during pregnancy and at delivery. If the dots are connected they represent longitudinally followed women and if isolated they represent different groups of women.

In 10 of the 11 studies postpartum malaria was compared with longitudinal data from women during pregnancy or at delivery (Additional file 2: Table S2). Three of these studies compared postpartum data with non-pregnant, non-postpartum women as well [17,19,22], whereas one study only used the latter as a control group[24]. Figure 2 shows the active screening regimen of each study.

The methods to describe post partum malaria varied in epidemiological indicators between studies. Distinction between primi- or multigravida was made in seven studies (Additional file 2: Table S2). Two studies conducted *P.falciparum *genotyping on delivery samples and postpartum samples[24,25], and sensitive detection for sub-microscopic infection at delivery and postpartum was performed in one study[25].

Chloroquine or sulphadoxine-pyrimethamine were used for treatment, chemoprophylaxis or IPTp, while resistance of *P.falciparum *parasites to these drugs was reported in six of these papers (Additional file 2: Table S2). In four of them, definitions about drug resistance were available, either in the text or by referring to other articles [17,23,25,26]. Self-treatment with anti-malarials was described in two studies[19,22]. Various treatment protocols of malaria episodes in pregnancy were used: in four studies any detected parasitaemia was treated regardless of symptoms, whereas in four other studies only women with symptomatic infections or high parasite counts were treated with anti-malarials (Additional file 2: Table S2).

### Main outcome of included studies

**Figure 3:**
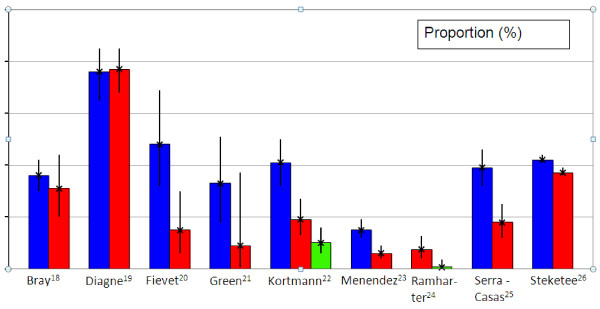
**Proportion of malaria positive women during pregnancy, postpartum or in non-pregnant, non-postpartum women**. Blue represents pregnant women, red represents post partum women and green represents non-pregnant, non-postpartum women.

The pertinent points relating to postpartum susceptibility from each of the 11 manuscripts are discussed in detail in Additional file 3. Nine of the 10 included studies that compared malaria during the postpartum period with malaria during pregnancy, showed a decline in the proportions of malaria positive women during the postpartum period (Figure 3, Additional file 3). The three studies that compared postpartum data with non- pregnant, non-postpartum controls found higher proportions in postpartum women compared to controls [19,22,24]. Of the seven studies that analysed malaria in the postpartum period separately for different gravidities, six studies observed more malaria episodes in primiparous postpartum women compared to multiparous women (RR calculated from 3 studies with proportions available [23,25,27] = 2.77 (95% CI 1.61 - 4.77), p < 0.001). Only the study from Gabon showed a non-significant difference in risk for primiparous compared to multiparous postpartum women (RR = 0.3 (95% CI 0.0 - 1.4), p = 0.12.)

### Main outcome of studies witht IPTp or chemoprophylaxis

In two of the three studies, where weekly chloroquine chemoprophylaxis was used during pregnancy and stopped at delivery, an increase in malaria after delivery was demonstrated. In PNG [17], the *P. falciparum *and *P. vivax *incidence per person-month increased until four months postpartum for all gravidae compared to pregnancy, and this was significant (p = 0.002) for multiparous women with *P. vivax*. Proportions could not be calculated. Also in Malawi [26], an increase in malaria after delivery was noticed, from 289/1494 (19%) at delivery to 1430/3864 (37%), at 2 months after delivery, p < 0.001. In Gabon [24] malaria data during pregnancy were not available and a comparison with postpartum data could not be made.

In Mozambique [23], the IPTp arm showed a decline in malaria proportions from 35/493 (7%) at delivery to 13/416 (3%) at two months after delivery, p = 0.004. By PCR method [25], the proportion of infected women at delivery was 38/187 (20%) compared to 27/138 (20%) at 8 weeks postpartum, p = 0.866.

### *Plasmodium falciparum *genotyping and sensitive detection

In Gabon [24], samples with PCR positive placental infections for the merozoite surface antigen-2 (*msa2*) [28] were compared to postpartum samples: five of the 16 puerperal malaria cases (31%, 95% CI 11-59%) carried the same falciparum genotype as detected in the placenta. In Mozambique [25], at eight weeks postpartum 50% (13/26) of women were infected with at least one parasite strain that was present at delivery, and detected with merozoite surface proteins (*msp1 *and *msp2*) [29]. In this study the prevalence of submicroscopic infections in the placebo and IPTp arm combined was 21% (73/352) at delivery and 13% (43/340) postpartum [25].

### Parasite density and symptoms

Five studies (four without IPTp or chemoprophylaxis [18-20,22], and one with chemoprophylaxis [26]) provided information about parasite densities. All reported lower densities during the postpartum period compared to pregnancy, two of which were significantly lower (both p = 0.02) [19,20].

Four studies provided information about symptoms. In the study from Senegal [19], all episodes that occurred during pregnancy, early postpartum (first 60 days) and in the control period were mild and rapidly cured. Few symptomatic postpartum malaria infections (defined as parasitaemia with a fever) were reported in the study from Mozambique (5.1%, (2/39) [23] and Cameroon (0/33) [20]. In Gabon [24], 61% (11/18) of malaria episodes in the postpartum group were symptomatic, versus 17% (1/6) in the control group. No study reported severe malaria in the postpartum period.

## Discussion

Nine of 10 reviewed studies suggested that the risk for malaria infection during pregnancy decreased after delivery, and one study suggests an equivalent risk after delivery compared to pregnancy. Postpartum women had more episodes of malaria than non-pregnant, non-postpartum controls in the three studies that included a control group.

However, the results of this review must be carefully interpreted, as the majority of studies were not designed to document specifically postpartum malaria, and there was a large variability in study designs and outcomes. The use of preventive drugs is an example of such a complicating factor: in more than half (6/11) of the studies IPTp or chemoprophylaxis were used during pregnancy. Chloroquine (weekly chemoprophylaxis) and sulphadoxine-pyrimethamine (IPTp) have a long half life and women are protected from malaria until the drug concentration falls below the minimum inhibitory concentration [30]. Depending on the timing of the last dose in pregnancy, drug half life and level of drug resistance, protection could be prolonged into the early postpartum period and may result in an underestimation of the postpartum susceptibility to malaria when compared to non pregnant non postpartum controls who did not use any prevention. In Mozambique, reduced prevalence of malaria parasitaemia at delivery and eight weeks postpartum (detected by malaria smear) was attributed to IPTp-sulphadoxine-pyrimethamine, though the last dose of IPTp was given at a mean time of 77 days before delivery [23]. This is much longer than the one-month estimated protective effect of sulphadoxine-pyrimethamine [21,30]. However, when PCR detection was used in a selection of women from the same study [25], it was reported that women in the IPTp group were indeed protected from malaria at delivery, but not anymore at eight weeks postpartum. Many post partum women harboured sub microscopic levels of parasites. Another effect of preventive anti-malarials during pregnancy was seen with use of weekly chemoprophylaxis. In 1985 when *P.vivax *was still sensitive to chloroquine in PNG, an increase in malaria incidence (*P. falciparum*, but especially *P. vivax*) was noticed in all gravidae as soon as the chloroquine chemoprophylaxis was stopped at delivery [17]. Such a "rebound effect" was noticed in a chemoprophylaxis study from Tanzania [26] as well as in children [31]. Chemoprophylaxis may have suppressed parasitaemia in pregnancy to sub-microscopic levels, but when it was discontinued at delivery, the parasites became microscopically detectable. This is in agreement with the recent studies, which were able to use PCR genotyping for detection of placental and postpartum parasites [24,25].

In the reviewed studies, postpartum women had more malaria episodes than non-pregnant, non-postpartum controls making plausible the existence of a transition phase, in which the susceptibility returned to pre-pregnancy levels. From the data available, the length of such a period could not be determined. In Senegal, the increased incidence went back to pre-pregnancy levels within 90 days after delivery [19], but in Tanzania women still had an increased prevalence compared to controls six months after delivery [22]. A mechanism to explain the decreased susceptibility in the post-partum period is maternal immunity. Fievet and colleagues observed an impairment in the IL-2 response, and unaffected or enhanced responses in IL-4 and interferon-gamma, in 33 women from Cameroon followed during their first pregnancy and at 6 months after delivery [20]. This was thought to favor a general depression of cellular immunity in pregnancy, rather than a specific malaria phenomenon. The transition phase from suppressed pregnancy immunity to normal pre-pregnancy levels has also been hypothesized by others [24]. Another mechanism to explain a transition phase is the persistence of malaria parasites from pregnancy due to inadequate treatment. Resistance of *P. falciparum *to chloroquine or sulphadoxine-pyrimethamine was mentioned in 55% (6/11) of articles. Ineffective treatments do not radically cure malaria infections [32], and these infections may recrudesce in the postpartum period. The only available *P.falciparum *genotyping data demonstrated that 30-50% of the postpartum infections were persistent from delivery until the postpartum period [24,25]. Hence, highly effective treatment during pregnancy, or IPTp regimens with highly effective drugs would impact significantly on reducing postpartum infection as well. In addition primiparous women have less antibodies to the CSA binding parasites that cause placental malaria [4] This could explain the increased risk of post partum malaria in primiparous women compared to multiparous mothers observed in six of seven studies reviewed.

To protect the post partum mother and her neonate [33] from malaria, an additional treatment dose of highly effective treatment at delivery may be considered as part of the IPTp strategy. Self-treatment may affect the presence of parasitaemia [34], but this was only reported in two manuscripts. Self-treatment effects are likely to vary with the quality of the drug, the dose and duration of treatment [35,36].

A limitation of this review is that in most published articles microscopy was used to detect malaria, whereas microscopy failed to detect 75% of the PCR detected parasitaemia during the postpartum period in a recent study [25]. The same study reports that 20 (77%) out of the 26 women with positive placenta-paired samples had not received treatment during labour because parasitaemia was sub-microscopic. The authors concluded that such untreated sub-microscopic infections persist until the postpartum period and reported a five-fold higher risk (95% CI 2.49-10.63) of *P. falciparum *infection eight weeks after delivery in women with sub-microscopic placental malaria. This relation between placental and postpartum malaria was not shown in, for example, the study from Senegal [19], where microscopy was used. However, at the same time, in the same area, but in the general population, high levels of sub-patent *P. falciparum *levels (two third of microscopically negative slides) were reported, using PCR [37]. Clearly, more investigation is needed to determine the burden of sub-microscopic malaria infections around delivery and in the post partum period.

There was an increased number of symptomatic infections in the postpartum period in Senegal [19] and Gabon [24]. Asymptomatic untreated (sub-microscopic) infections in pregnancy may have become symptomatic after delivery, or asymptomatic episodes were detected and reported as symptomatic due to accompanying fever, as women in the postpartum period are prone to febrile co-morbidities such as mastitis and endometritis. In both studies, the incidence of asymptomatic malaria episodes did not increase as much as for symptomatic episodes.

Data on non-falciparum species and the risk of postpartum infection has only been described for PNG [17]. At least for *P. vivax *and *P. ovale *it would be very useful to know more about the postpartum risks for infection as primaquine, the only available drug for eradication of the liver stage of these species, cannot be used during pregnancy. Postpartum detection may provide a window of opportunity for primaquine therapy so that these species do not relapse in subsequent pregnancies.

## Conclusions

Nine of 10 studies demonstrated a decreasing risk of malaria after pregnancy, but some suggested a risk higher than non-pregnant, non-postpartum controls. A transition phase in which the susceptibility returns to pre-pregnancy levels seems plausible, but from the data available the length of this period could not be determined. Due to methodological differences in the currently available literature the results of this review have to be carefully interpreted. Future studies that precisely document the epidemiology and treatment of malaria episodes during pregnancy, drug resistance, timing of IPTp doses, and self-treatment are needed. Recommendations for the design of a postpartum malaria study are given in Additional file 4.

Sensitive detection for submicroscopic and genotyping of parasites during pregnancy and post partum would be an important component of future studies. The limited data available implies that efforts to detect and treat malaria effectively during pregnancy [38] will prevent women from entering the postpartum period with residual parasitaemia. Administration of an additional treatment dose of an effective drug at delivery to all pregnant women in endemic areas may provide some health benefits to the mother (less anaemia), although be of no direct consequence for the newborn, except in reducing the risk of congenital malaria [33]. Health workers need to be mindful that malaria acquired during pregnancy can have consequences in the postpartum period.

## Competing interests

The authors declare that they have no competing interests.

## Authors' contributions

MB searched for and identified the included studies, extracted data on an Excel spreadsheet and drafted the manuscript. MR searched for and included the studies and helped to draft the manuscript. BB searched for articles, provided raw data, and helped to draft the manuscript. RM did the eligibility assessment and extracted data on an Excel spreadsheet and helped to draft the manuscript. FN did the eligibility assessment and helped to draft the manuscript. All authors read and approved the final manuscript.

## Supplementary Material

Additional file 1 **Table S1**. Characteristics of the studies included in the review on postpartum malaria. Click here for file

Additional file 2 **Table S2**. Quality measures of the included studies. Click here for file

Additional file 3 **Summary of the pertinent points relating to postpartum susceptibility from 11 manuscripts in alphabetic order of the first author**. Click here for file

Additional file 4 **Recommendations for the design of a postpartum malaria study**. Click here for file

## References

[B1] Wickramasuriya GAW Some observations on malaria occurring in association with pregnancy J Obstet Gynaecol 1935 42 816 834

[B2] Desai M ter Kuile FO Nosten F McGready R Asamoa K Brabin B Newman RD Epidemiology and burden of malaria in pregnancy Lancet Infect Dis 2007 7 93 104 10.1016/S1473-3099(07)70021-X 17251080

[B3] McGready R Lee S Wiladphaingern J Ashley E Rijken M Boel M Simpson J Paw M Pimanpanarak M Mu O Singhasivanon P White N Nosten F Adverse effects of falciparum and vivax malaria and the safety of antimalarial treatment in early pregnancy: a population-based study Lancet Infect Dis 2011 10.1016/S1473-3099(11)70339-5 PMC3346948 22169409

[B4] Fried M Nosten F Brockman A Brabin BJ Duffy PE Maternal antibodies block malaria Nature 1998 395 851 852 10.1038/27570 9804416

[B5] WHO A Strategic Framework for Malaria Control During Pregnancy in the African Region 2011 http://www.rollbackmalaria.org/toolbox/tool_MalariaPreventionInPregnancy.html

[B6] Vallely L Ahmed Y Murray SF Postpartum maternal morbidity requiring hospital admission in Lusaka Zambia - a descriptive study. BMC Pregnancy Childbirth 2005 5 1 10.1186/1471-2393-5-1 PMC549039 15686592

[B7] Barnett S Nair N Tripathy P Borghi J Rath S Costello A A prospective key informant surveillance system to measure maternal mortality - findings from indigenous populations in Jharkhand and Orissa India BMC Pregnancy Childbirth 2008 8 6 10.1186/1471-2393-8-6 PMC2268911 18307796

[B8] Nguyen-Dinh P Steketee RW Greenberg AE Wirima JJ Mulenda O Williams SB Rapid spontaneous postpartum clearance of *Plasmodium falciparum *parasitaemia in African women Lancet 1988 2 751 752 2901606 10.1016/s0140-6736(88)90229-2

[B9] Bottero J Briand V Agbowai C Doritchamou J Massougbodji A Cot M Spontaneous postpartum clearance of *Plasmodium falciparum *parasitemia in pregnant women, Benin Am J Trop Med Hyg 2011 84 267 269 10.4269/ajtmh.2011.10-0139 21292897 PMC3029180

[B10] Msyamboza KP Savage EJ Kazembe PN Gies S Kalanda G D'Alessandro U Brabin BJ Community-based distribution of sulfadoxine-pyrimethamine for intermittent preventive treatment of malaria during pregnancy improved coverage but reduced antenatal attendance in southern Malawi Trop Med Int Health 2009 14 183 189 10.1111/j.1365-3156.2008.02197.x 19207178

[B11] Lindsay S Ansell J Selman C Cox V Hamilton K Walraven G Effect of pregnancy on exposure to malaria mosquitoes Lancet 1972 2000 355 10.1016/S0140-6736(00)02334-5 10859048

[B12] Riley EM Schneider G Sambou I Greenwood BM Suppression of cell-mediated immune responses to malaria antigens in pregnant Gambian women Am J Trop Med Hyg 1989 40 141 144 2493200 10.4269/ajtmh.1989.40.141

[B13] Sholapurkar SL Mahajan RC Gupta AN Wangoo A Cellular immunity in pregnant and non-pregnant women with malarial infection Asia Oceania J Obstet Gynaecol 1990 16 27 32 2344308 10.1111/j.1447-0756.1990.tb00211.x

[B14] WHO:World Health Organization Postpartum care of mother and newborn: a practical guide Geneva 1998 10655832

[B15] Malaria in Pregnancy Consortium http://www.mip-consortium.org

[B16] Liberati A Altman DG Tetzlaff J Mulrow C Gotzsche PC Ioannidis JP Clarke M Devereaux PJ Kleijnen J Moher D The PRISMA statement for reporting systematic reviews and meta-analyses of studies that evaluate healthcare interventions: explanation and elaboration BMJ 2009 339 b2700 10.1136/bmj.b2700 19622552 PMC2714672

[B17] Brabin BJ Ginny M Alpers M Brabin L Eggelte T Van der Kaay HJ Failure of chloroquine prophylaxis for falciparum malaria in pregnant women in Madang, Papua New Guinea Ann Trop Med Parasitol 1990 84 1 9 2184784 10.1080/00034983.1990.11812428

[B18] Bray RS Anderson MJ Falciparum malaria and pregnancy Trans R Soc Trop Med Hyg 1979 73 427 431 10.1016/0035-9203(79)90170-6 400205

[B19] Diagne N Rogier C Sokhna CS Tall A Fontenille D Roussilhon C Spiegel A Trape JF Increased susceptibility to malaria during the early postpartum period N Engl J Med 2000 343 598 603 10.1056/NEJM200008313430901 10965006

[B20] Fievet N Cot M Ringwald P Bickii J Dubois B Le Hesran JY Migot F Deloron P Immune response to *Plasmodium falciparum *antigens in Cameroonian primigravidae: evolution after delivery and during second pregnancy Clin Exp Immunol 1997 107 462 467 10.1046/j.1365-2249.1997.d01-966.x 9067518 PMC1904608

[B21] Green MD van Eijk AM van Ter Kuile FO Ayisi JG Parise ME Kager PA Nahlen BL Steketee R Nettey H Pharmacokinetics of sulfadoxine-pyrimethamine in HIV-infected and uninfected pregnant women in Western Kenya J Infect Dis 2007 196 1403 1408 10.1086/522632 17922406

[B22] Kortmann HF Malaria and Pregnancy 1972 Amsterdam: University of Amsterdam

[B23] Menendez C Bardaji A Sigauque B Romagosa C Sanz S Serra-Casas E Macete E Berenguera A David C Dobano C Naniche D Mayor A Ordi J Mandomando I Aponte JJ Mabunda S Alonso PL A randomized placebo-controlled trial of intermittent preventive treatment in pregnant women in the context of insecticide treated nets delivered through the antenatal clinic PLoS One 2008 3 e1934 10.1371/journal.pone.0001934 18398460 PMC2277457

[B24] Ramharter M Grobusch MP Kiessling G Adegnika AA Moller U Agnandji ST Kramer M Schwarz N Kun JF Oyakhirome S Issifou S Borrmann S Lell B Mordmuller B Kremsner PG Clinical and parasitological characteristics of puerperal malaria J Infect Dis 2005 191 1005 1009 10.1086/427781 15717279

[B25] Serra-Casas E Menendez C Dobano C Bardaji A Quinto L Ordi J Sigauque B Cistero P Mandomando I Alonso PL Mayor A Persistence of *Plasmodium falciparum *parasites in infected pregnant Mozambican women after delivery Infect Immun 2011 79 298 304 10.1128/IAI.00814-10 21041485 PMC3019917

[B26] Steketee RW Wirima JJ Bloland PB Chilima B Mermin JH Chitsulo L Breman JG Impairment of a pregnant woman's acquired ability to limit *Plasmodium falciparu *by infection with human immunodeficiency virus type-1 Am J Trop Med Hyg 1996 55 42 49 8702036 10.4269/ajtmh.1996.55.42

[B27] Watkinson M Rushton DI Plasmodial pigmentation of placenta and outcome of pregnancy in West African mothers Br Med J (Clin Res Ed) 1983 287 251 254 10.1136/bmj.287.6387.251 PMC1548862 6409270

[B28] Ntoumi F Contamin H Rogier C Bonnefoy S Trape JF Mercereau-Puijalon O Age-dependent carriage of multiple *Plasmodium falciparum *merozoite surface antigen-2 alleles in asymptomatic malaria infections Am J Trop Med Hyg 1995 52 81 88 7856831 10.4269/ajtmh.1995.52.81

[B29] Mayor A Serra-Casas E Bardaji A Sanz S Puyol L Cistero P Sigauque B Mandomando I Aponte JJ Alonso PL Menendez C Sub-microscopic infections and long-term recrudescence of *Plasmodium falciparu *in Mozambican pregnant women Malar J 2009 8 9 10.1186/1475-2875-8-9 19134201 PMC2633011

[B30] White NJ Intermittent presumptive treatment for malaria PLoS Med 2005 2 e3 10.1371/journal.pmed.0020003 15696210 PMC545196

[B31] Greenwood BM David PH Otoo-Forbes LN Allen SJ Alonso PL Armstrong Schellenberg JR, Byass P, Hurwitz M, Menon A, Snow RW: Mortality and morbidity from malaria after stopping malaria chemoprophylaxis Trans R Soc Trop Med Hyg 1995 89 629 633 10.1016/0035-9203(95)90419-0 8594677

[B32] McGready R White NJ Nosten F Parasitological efficacy of antimalarials in the treatment and prevention of falciparum malaria in pregnancy 1998 to 2009: a systematic review BJOG 2011 118 123 135 10.1111/j.1471-0528.2010.02810.x 21159117

[B33] Poespoprodjo JR Fobia W Kenangalem E Hasanuddin A Sugiarto P Tjitra E Anstey NM Price RN Highly effective therapy for maternal malaria associated with a lower risk of vertical transmission J Infect Dis 2011 204 1613 1619 10.1093/infdis/jir558 21908728 PMC3192188

[B34] Hodel EM Kabanywanyi AM Malila A Zanolari B Mercier T Beck HP Buclin T Olliaro P Decosterd LA Genton B Residual antimalarials in malaria patients from Tanzania-implications on drug efficacy assessment and spread of parasite resistance PLoS One 2009 4 e8184 10.1371/journal.pone.0008184 20011529 PMC2788605

[B35] Atemnkeng MA De Cock K Plaizier-Vercammen J Quality control of active ingredients in artemisinin-derivative antimalarials within Kenya and DR Congo Trop Med Int Health 2007 12 68 74 17207150 10.1111/j.1365-3156.2006.01769.x

[B36] Newton PN McGready R Fernandez F Green MD Sunjio M Bruneton C Phanouvong S Millet P Whitty CJ Talisuna AO Proux S Christophel EM Malenga G Singhasivanon P Bojang K Kaur H Palmer K Day NP Greenwood BM Nosten F White NJ Manslaughter by fake artesunate in Asia-will Africa be next? PLoS Med 2006 3 e197 10.1371/journal.pmed.0030197 16752952 PMC1475657

[B37] Bottius E Guanzirolli A Trape JF Rogier C Konate L Druilhe P Malaria: even more chronic in nature than previously thought; evidence for subpatent parasitaemia detectable by the polymerase chain reaction Trans R Soc Trop Med Hyg 1996 90 15 19 10.1016/S0035-9203(96)90463-0 8730301

[B38] WHO Guidelines for the treatment of malaria 2010 2 Geneva: WHO 25473692

