# Supplementary material for: The epidemiology of postpartum malaria: a systematic review
Source: Malar J. 2012 Apr 13;11:114. doi: 10.1186/1475-2875-11-114 (PMC3379929; doi:10.1186/1475-2875-11-114)
Supplement: Additional file 1 — Table S1. Characteristics of the studies included in the review on postpartum malaria. [file 1475-2875-11-114-S1.DOC]

Additional Table 1. Characteristics of the studies included in the review on postpartum malaria.

| **Author** | **Country** | **Year** | **Sampling during pregnancy** | **Sampling after delivery** | **Primary subject under study** | **Sample size** | **Transmission** | **Species analysed** | **Treatment** |
| --- | --- | --- | --- | --- | --- | --- | --- | --- | --- |
| Brabin[17] | PNG | 1985-1987 | Weekly | Monthly for 4 months | Chemoprophylaxis | 620 | High | Pf , Pv, Pm | CQ and SP |
| Bray[18] | The Gambia | 1977 | Once in late pregnancy | Once in the 1^st^ month | Immunity | 332 | Seasonal | Pf | nm |
| Diagne[19] | Senegal | 1990-1998 | Monthly | Monthly for 1 year | Epidemiology | 38 | High | Pf | nm |
| Fievet[20] | Cameroon | 1992 | Once at 6 months | Once at 6 months | Immunity | 33 | Hyperendemic perennial | Pf | nm |
| Green[21] | Kenya | 1999-2000 | Once at EGA 16-28 weeks | Once around 2 months | Pharmacokinetics | 33 | nm | nm | nm |
| Kortmann[22] | Tanzania | 1968-1969 | At least monthly | At least monthly for 7 months | Immunity | 26 | Perennial* | Pf | CQ |
| Menendez[23] | Mozambique | 2003-2005 | At delivery | Once at 2 months | IPTp | 1030 | Perennial | Pf | CQ or SP |
| Ramharter[24] | Gabon | 2003-2004 | No samples in pregnancy | Weekly for 10 weeks | Puerperal malaria | 299 | High | Pf | Q for Pf, CQ for Pm, Po |
| Serra-Casas[25] | Mozambique | 2003-2005 | At delivery | Once at 2 months | Malaria postpartum | 402 | Perennial* | Pf | CQ and SP |
| Steketee[26] | Malawi | 1987-1989 | At inclusion and at delivery | Every 2 months for 1 year | Chemoprophylaxis | 2946 | High | Pf | CQ |
| Watkinson[27] | The Gambia | 1979-1980 | At least every 6 weeks | Once before 18 months | Placental malaria | 95 | High | Pf | CQ |

*With seasonality. IPTp intermittent preventive treatment in pregnancy, PNG Papua New Guinea, Pf *Plasmodium falciparum*, Pm *Plasmodium malariae*, Po *Plasmodium Ovale*, Pv *Plasmodium vivax*, nm not mentioned, CQ chloroquine, Q quinine, SP sulphadoxine-pyrimethamine
