# Supplementary material for: The epidemiology of postpartum malaria: a systematic review
Source: Malar J. 2012 Apr 13;11:114. doi: 10.1186/1475-2875-11-114 (PMC3379929; doi:10.1186/1475-2875-11-114)
Supplement: Additional file 2 — Table S2. Quality measures of the included studies. [file 1475-2875-11-114-S2.DOC]

Additional Table 2. Quality measures of the included studies.

| **Author** | **Controls** | **Prevention in pregnancy** | **Parity analysed separately** | **Indicators** | **Passive or active screening** | **Where screening** | **Lost to follow up (%)** | **Treatment of asymptomatic parasitaemia during pregnancy** | **Drug resistance** | **Screening for self treatment** |
| --- | --- | --- | --- | --- | --- | --- | --- | --- | --- | --- |
| Brabin[17] | Unmatched^ | Chemo  prophylaxis | Y | Prevalence and incidence | active | ANC and same study group after delivery | nm | Y | Y | N |
| Bray[18] | Nil | None | N | Prevalence | active | ANC and PNC | na | nm | nm | N |
| Diagne[19] | Matched | None | Y | Incidence | both | Village based | nm | N | Y | Y |
| Fievet[20] | Nil | None | Only G1 | Prevalence | active | ANC and same study group after delivery | 0/33 | nm | nm | N |
| Green[21] | Nil | IPTp | Only G1 and G2 | Proportion | active | ANC and same study group after delivery | 22/33 (67%) | Y | Y | N |
| Kortmann[22] | Unmatched | none | N | Parasite rate | both | ANC and same study group after delivery | nm | N | N | Y |
| Menendez[23] | Nil | IPTp | Y | Prevalence | both | ANC and same study group after delivery | 63/495 (13%) | N | Y | N |
| Ramharter[24] | Matched | Chemo  prophylaxis | Y | Incidence | both | Study group included at delivery | 15/299 (5%) | nm | nm | N |
| Serra-Casas[25] | Nil | IPTp | Y | Prevalence | both | ANC and same study group after delivery | 48/402 (12%) | Y | Y | N |
| Steketee[26] | Nil | Chemo  prophylaxis | Y | Prevalence* | both | ANC and same study group after delivery | nm | N | Y | N |
| Watkinson[27] | Nil | none | Y | Incidence | both | ANC, open door clinic and PNC | na | Y | nm | N |

*After delivery a combination of cross-sectional surveys was used. ^ A control group was present in this study, but it was not possible to retrieve the raw data to calculate cumulative proportions. N No, Y yes, nm not mentioned, na not applicable, IPTp Intermittent preventive treatment in pregnancy, ANC antenatal care, PNC postnatal care
