# Supplementary material for: The epidemiology of postpartum malaria: a systematic review
Source: Malar J. 2012 Apr 13;11:114. doi: 10.1186/1475-2875-11-114 (PMC3379929; doi:10.1186/1475-2875-11-114)
Supplement: Additional file 3 — Summary of the pertinent points relating to postpartum susceptibility from 11 manuscripts in alphabetic order of the first author. [file 1475-2875-11-114-S3.DOC]

Additional file 1

Summary of the pertinent points relating to postpartum susceptibility from 11 manuscripts in alphabetic order of the first author.

Brabin *et al*, Papua New Guinea[17]

In PNG, malaria data during pregnancy at the first antenatal visit (before using chloroquine prophylaxis) showed a higher prevalence of malaria (42.6% for primigravida and 24.3% for multigravida) than during the 4 months postpartum when it was 20.4% *(P.falciparum)* and 7.8% *(P.vivax)* for primipara, and 17.5% and 5.3% for multipara, respectively. Parasite rates of a control group of non pregnant women were reported, but it was not possible to retrieve the raw data to calculate proportions for comparison of malaria in postpartum women with pregnant women and controls.

Bray and Anderson, The Gambia[18]

In the Gambia in 1977 when malaria prophylaxis was not part of the national policy yet, the proportion of infected women was higher in the last 2 months of pregnancy (98/274 (35.8%)) than in the first month after delivery (18/58 (31.0%) p=0.49).

Diagne *et al*, Senegal[19]

In Senegal, a cohort of 38 women was closely followed for eight years through 71 pre-conceptual, pregnant, and postpartum periods to study postpartum malaria. Pre-pregnancy data were used as control data. Chemoprophylaxis or IPTp were intentionally not provided to pregnant or postpartum women in this study, with the ethical justification of semi-immunity in the population, the high level of chloroquine resistance, and daily visits were provided to all study participants for recording of symptoms. The study reported an adjusted relative risk compared to the pre-pregnancy status for “clinical malaria” (defined as any case of fever (axillary temperature of at least>37.5°C) or fever-related symptoms (headache, vomiting, and a subjective sensation of fever) associated with a ratio of parasites to leucocytes that exceeds an age-dependent pyrogenic threshold previously identified) of 1.0 (95% CI 0.3–3.0, p= 0.97) in the first trimester, 2.8 (95% CI 1.1-7.4, p= 0.04) in the second, 3.1(95% CI 1.2-7.9, p= 0.02) in the third trimester of pregnancy and 4.1 (95%CI 1.8-9.5, p= 0.001) in the 60 days postpartum, indicating that the risk for symptomatic malaria postpartum was higher than during pregnancy. Postpartum women did not have more asymptomatic parasitaemias than pregnant women as the adjusted odds ratio compared to before pregnancy for *P.falciparum* parasitaemia was 2.2 (95% CI 1.5-3.2, p= <0.001), 2.5 (95% CI 1.7-3.6, p= 0<0.001) and 2.1(95% CI1.4-3.1, p= <0.001) in the first, second and third trimester, compared to 1.8 (95% CI 1.1-2.7, p= 0.014) in the postpartum period. The susceptibility to malaria returned to pre-pregnancy levels 90 days after delivery. No relation was found between placental malaria detected by malaria smear and postpartum malaria. When crude numbers were analysed (after contacting an author), the proportions of women that had any parasitaemia during the 1^st^, second and third trimester were 42/53 (79.2%), 47/61 (77.0%) and 48/63 (76.2%) respectively, all not significantly different compared to the proportion of women with malaria in the first 3 months postpartum; 65/84 (77.4%), p= 0.80, 0.96 and 0.87 respectively. This is the only study (without use of IPTp or chemoprophylaxis) that does not show a decline in the proportion of women infected with malaria after delivery.

Fievet *et al*, Cameroon[20]

In Cameroon, the proportion of infected primigravida was higher at the sixth month of pregnancy (16/33 (48.5%)) than it was in the same women at six months postpartum (5/33 (15.1%), p=0.02).

Green *et al*, Kenya[21]

In Kenya, 33 women were tested for parasitaemia before the administration of IPTp in the 16-28^th^ week of pregnancy in a pharmacokinetic study. This procedure was repeated at 2 months postpartum with 11 of the 33 women. The proportion of positive women was higher during pregnancy (11/33 (33.3%)) than it was after delivery (1/11 (9.1%), p=0.09).

Kortmann, Tanzania[22]

In a study in Tanzania, a control group of 153 non-pregnant persons (women and men) age 20-39 years was screened for malaria during a survey in the same area and time period[28] as a cohort study of 26 unprotected pregnant women. In this study the proportion of women with malaria at several screenings during pregnancy was 49/120 (41%), which was higher than the proportion measured at several time points during the first to sixth months following delivery: 22/116 (19%), p<0.001. The author concluded that the parasite rate during pregnancy was about twice as high as it was after delivery, and that the changes from pregnant to non-pregnant values seemed to occur within a week after delivery, as directly the first screening after delivery showed this decline. The proportion of infected women after delivery was higher than the proportion of malaria infected people in the control group: 15/153 (10%), p=0.03.

Menendez *et al,* Mozambique[23]

In a RCT in Mozambique, the proportion in the placebo arm of women with malaria was higher at delivery (75/495 (15.2%)) than it was two months after delivery 26/432 (6.0%), p<0.001.

Ramharter *et al,* Gabon[24]

In a study in Gabon, conducted to investigate puerperal malaria, the proportion of clinical malaria (defined as >100 *P.falciparum* parasites/µl, fever or a history of fever or presence of other symptoms associated with malaria) in postpartum women was nearly ten times higher (11/148 (7.4%)) than it was in non-pregnant, non-postpartum women matched for age and location (1/136 (0.7%), p=0.005). The rate ratio for malaria in the postpartum period compared to controls based on the incidences given in the paper was 9.8 (95% CI 1.4-420, p = 0.006)[24]. The rate ratio for asymptomatic infections postpartum compared to controls was 2.7 (95% CI 1.0-8.2, p= 0.03). Matched pair analysis showed no relationship between the presence of microscopically detected placental parasites and the development of parasitaemia postpartum. A high proportion, 77% (114/148), of puerperal women reported use of chloroquine prophylaxis during pregnancy but data about chloroquine intake in the previous month before inclusion was not available for control women. The authors conclude the use of chloroquine prophylaxis was not significantly associated with the development of *P.falciparum* during postpartum follow-up (RR 0.4 with 95% CI 0.2-1.5, p= 0.14) compared to controls.

Serra-Casas *et al*, Mozambique[25]

In another study from Mozambique, PCR methods were used to answer the question whether malaria infections during the postpartum period had their origin during pregnancy. The proportion of malaria positive women at delivery in the placebo arm was 67/172 (39%) compared to 24/133 (18%) at 8 weeks postpartum (p<0.001).

Steketee *et al*, Malawi[26]

In Malawi, women were enrolled in four different malaria prevention groups during antenatal care. The proportion of malaria positive women at inclusion, before any drug administration, was 1142/2694 (42%) compared to a proportion from screens around 2 months after delivery of 1430/3864 (37%), p<0.001.

Watkinson and Rushton, The Gambia[27]

A study in The Gambia showed that malaria was 3.8 times more prevalent (p= 0.001) in pregnant than in lactating women, based on malaria smears from pregnant women visiting antenatal clinics and lactating women visiting postnatal clinics. Proportions could not be calculated as the raw data were not available.
