# Supplementary material for: The epidemiology of postpartum malaria: a systematic review
Source: Malar J. 2012 Apr 13;11:114. doi: 10.1186/1475-2875-11-114 (PMC3379929; doi:10.1186/1475-2875-11-114)
Supplement: Additional file 4 — Recommendations for the design of a postpartum malaria study. [file 1475-2875-11-114-S4.DOC]

Additional file 4. Recommendations for the design to study postpartum malaria.

• A prospective cohort study starting in early pregnancy until at least 6 months postpartum where women are matched (age, residential location, parity) to non-pregnant, non-postpartum control women, followed-up at the same time to prevent seasonal bias.

• Similar diagnostics should be used during pregnancy, delivery, postpartum and in controls to detect malaria parasites. Real- time PCR would be ideal but when not available, microscopy or rapid detection tests would suffice with PCR specimens to be examined later.

• IPTp or chemoprophylaxis should preferably not be given during pregnancy, but regular screening and early treatment provided to all patients. If this is not feasible, similar preventive strategies should be provided for both study groups.

• Any parasitaemia (including asymptomatic) should be treated with the aim to cure radically. The treatment used in pregnant and non-pregnant women should be the same drug and the drugs should be equally efficacious in pregnant and non-pregnant women.

• PF genotyping of all infections (including submicroscopic) should enable the researcher to distinguish between new and recrudescent infections in both study groups.

• PV genotyping may not allow all infections to be genotyped but it would allow the proportion of reappearing infections which are homologous to be quantified.
